# To be or not to be tetraploid—the impact of marker ploidy on genomic prediction and GWAS of potato

**DOI:** 10.3389/fpls.2024.1386837

**Published:** 2024-07-30

**Authors:** Trine Aalborg, Kåre Lehmann Nielsen

**Affiliations:** ^1^ Department of Chemistry and Bioscience, Aalborg University, Aalborg, Denmark; ^2^ Research and Development, Kartoffelmelcentralen (KMC) Amba, Brande, Denmark

**Keywords:** *Solanum tuberosum*, genomic prediction, GBLUP, GWAS, potato breeding, tetraploid, genotyping, allele dosage

## Abstract

Cultivated potato, *Solanum tuberosum L*., is considered an autotetraploid with 12 chromosomes with four homologous phases. However, recent evidence found that, due to frequent large phase deletions in the genome, gene ploidy is not constant across the genome. The elite cultivar “Otava” was found to have an average gene copy number of 3.2 across all loci. Breeding programs for elite potato cultivars rely increasingly on genomic prediction tools for selection breeding and elucidation of quantitative trait loci underpinning trait genetic variance. These are typically based on anonymous single nucleotide polymorphism (SNP) markers, which are usually called from, for example, SNP array or sequencing data using a tetraploid model. In this study, we analyzed the impact of using whole genome markers genotyped as either tetraploid or observed allele frequencies from genotype-by-sequencing data on single-trait additive genomic best linear unbiased prediction (GBLUP) genomic prediction (GP) models and single-marker regression genome-wide association studies of potato to evaluate the implications of capturing varying ploidy on the statistical models employed in genomic breeding. A panel of 762 offspring of a diallel cross of 18 parents of elite breeding material was used for modeling. These were genotyped by sequencing and phenotyped for five key performance traits: chipping quality, length/width ratio, senescence, dry matter content, and yield. We also estimated the read coverage required to confidently discriminate between a heterozygous triploid and tetraploid state from simulated data. It was found that using a tetraploid model neither impaired nor improved genomic predictions compared to using the observed allele frequencies that account for true marker ploidy. In genome-wide associations studies (GWAS), very minor variations of both signal amplitude and number of SNPs supporting both minor and major quantitative trait loci (QTLs) were observed between the two data sets. However, all major QTLs were reproducible using both data sets.

## 1 Introduction

The cultivated tetraploid potato, *Solanum tuberosum L.*, is the world’s third most important food crop for global human consumption (FAOSTAT, 2024) and is of principal interest for future global food security (Devaux et al., 2014), as elite cultivars can be grown in diverse climate regions, and it is the highest yielding food crop (Haverkort and Struik, 2015). Most commercially grown cultivars are highly heterozygous autotetraploids (2*n* = 4*x* = 48) with four homologues of 12 chromosomes. This complicates breeding, because of the complex, tetrasomic inheritance of key performance traits (Ortiz and Mihovilovich, 2019) such as yield and other quantitative traits. Consequences include ineffective purging of deleterious alleles from populations and their accumulation in breeding clones (Pham et al., 2017), leading to signs of acute inbreeding depression in potatoes (Zhang et al., 2019), as well as arduous fixation of the desirable, recessive alleles underpinning improved trait phenotype in a single breeding line (Muthoni et al., 2015). Recently, the complexities of potato genetics have been further increased. In their chromosome-scale haplotype-resolved genome assembly of the tetraploid cultivar “Otava,” Sun et al. (2022) found that this elite potato cultivar was in fact not tetraploid across all loci (only 54% of genes) but rather presented with an average of 3.2 copies per gene and possible allelic gene copies of 1, 2, 3, and 4. This is caused by the frequent occurrence of large deletions in one or more of the phases, which often encompasses several genes. Missing alleles were similarly detected in assemblies of six other tetraploid cultivars, where the orthologous genes identified using a pan-genomics approach displayed an average of 2.65 copies per tetraploid (Hoopes et al., 2022). With irregular gene copy numbers, issues such as haploinsufficiency can become more prevalent compared to assumptions of an absolute tetraploid as gene expression correlates with both gene copy number and allele copy number (Sun et al., 2022). Extrapolating this argument, it is unknown how genomic regions with lower gene ploidy coincide with areas of segregation distortion and how/if variations in allele copy numbers are maintained during sexual crossing.

SNP array or sequence-based genotyping data is usually converted into allele dosages using a fixed tetraploid model and used by breeders and researchers to predict breeding values using genomic selection or identify trait quantitative trait loci (QTLs) using genome-wide association studies (GWAS) (Stich and Van Inghelandt, 2018; Selga et al., 2021; Wilson et al., 2021). Obviously, when >40% of all loci (extrapolating from “Otava”) are likely not tetraploid, this is not a true representation of the underlying genetic architecture and thus constitutes a formal systematic error, which might impact statistical models such as genomic prediction (GP) or GWAS. Furthermore, estimation of allele dosage in autopolyploids is notoriously challenging as the number of genotypes increases with ploidy, hence escalating the complexity of correctly assigning heterozygous allele dosages, eventually leading to misclassification issues (de Bem Oliveira et al., 2019; Yadav et al., 2024). Because high read depth is required to confidently call heterozygous classes (60–80× for tetraploids) (Uitdewilligen et al., 2013), another approach in polyploids is pseudo-diploidization, where all heterozygous states are concatenated into a single diploidized heterozygous class, for example, for an autotetraploid the simplex (AAAB), duplex (AABB), and triplex (ABBB) are represented by a single diploid class AB (Matias et al., 2019). In potato, such pseudo-diploidization resulted in a consistent deflation of prediction accuracy of 0.13 on average compared to using tetraploid genotypes (Endelman et al., 2018) with a trait-dependent response (Gemenet et al., 2020), indicating that the method of allele dosage estimation impacts prediction performance. Collapsing the heterozygosity also fails to capture gene level variation of dosage values, similar to using a tetraploid model.

The effect of various genotype classifications on GP and GWAS have previously been assessed in polyploid crop plants, including potatoes, sweet potatoes, tropical forages, blueberries, chrysanthemum, and sugar cane (Grandke et al., 2016; Endelman et al., 2018; de Bem Oliveira et al., 2019; Matias et al., 2019; de Bem Oliveira et al., 2020; Gemenet et al., 2020; Ferrão et al., 2021; Yadav et al., 2024). This includes using continuous genotypes alternative to discrete allele dosages, that is, using extracted allele frequencies directly from the observed genotype data (Ashraf et al., 2016), effectively reducing computational time by circumventing genotype calling steps. This approach is ideal for species with complex or poorly defined ploidy levels, and the application of continuous genotypes might improve the accuracy of GP and GWAS results by including a more realistic representation of the true genotype classes (de Bem Oliveira et al., 2019; Yadav et al., 2024). Continuous genotypes have been successfully used in GWAS of hexaploid chrysanthemum with comparable results to discrete genotype classes (Grandke et al., 2016). In sugar cane, a genetically highly complex crop with varying ploidy, continuous genotypes have been used to capture true allele dosage, compared to traditional strategies of pseudo-diploidization, and results showed trait-dependent responses in GP accuracy (Yadav et al., 2024). Similarly, a trait-dependent response to allele dosage strategies was found in GP of autotetraploid blueberry (de Bem Oliveira et al., 2019), while low-mid sequencing depth (6–12×) was sufficient to obtain prediction accuracies comparable to those using high-depth (60×) (de Bem Oliveira et al., 2020). Employing ratio genotypes, rather than calling discrete heterozygous genotypes that require high-sequencing depths, estimated 60–80× in tetraploids (Uitdewilligen et al., 2013), indicates that high-accuracy genomic models can be implemented in polyploids without substantial increases in genotyping costs (Ferrão et al., 2021).

While continuous genotypes have been evaluated in other crop plants, previous studies of potatoes have not included comparisons with between parametrized genotypes, either tetraploid or diploidized, and continuous genotypes (Endelman et al., 2018). However, allele frequency ratio genotypes have led to useful results in tetraploid potato (Sverrisdóttir et al., 2017, 2018; Aalborg et al., 2024). Prompted by the discovery of gene-level ploidy variation in potato, this raises the question of whether calling tetraploid SNPs across the genome, as commonly practiced, influences the results of genomic prediction and GWAS statistical models in potato, as this introduces genotype misclassifications across all non-tetraploid loci. To assess whether using the observed SNP ratio as a measure of ploidy at each SNP locus in the genome, that is, using continuous genotypes, affects model performance relative to calling tetraploid SNPs at all loci, we have compared the performance of standard additive GBLUP GP model and single-marker regression GWAS on a set of ~31k SNPs genotyped by GBS (genotyping-by-sequencing) of a 762-clone panel, called MASPOT, that were called as (1) observed variant allele frequencies (continuous) or (2) tetraploid at all loci. Screening five single key performance traits of varying genetic architectures (chipping quality, length/width ratio, senescence, dry matter content, and yield), we report on the effect of true SNP ploidy on the models commonly used in augmenting potato breeding and trait QTL characterization with the purpose of providing directives for future accommodation to the true ploidy of elite potato cultivars. Furthermore, we explore the practical possibility of resolving the ploidy status for each individual locus directly from sequencing data using simulated data.

## 2 Results and discussion

### 2.1 Triploid and tetraploid SNP status cannot be discriminated with confidence

**Figure 1:**
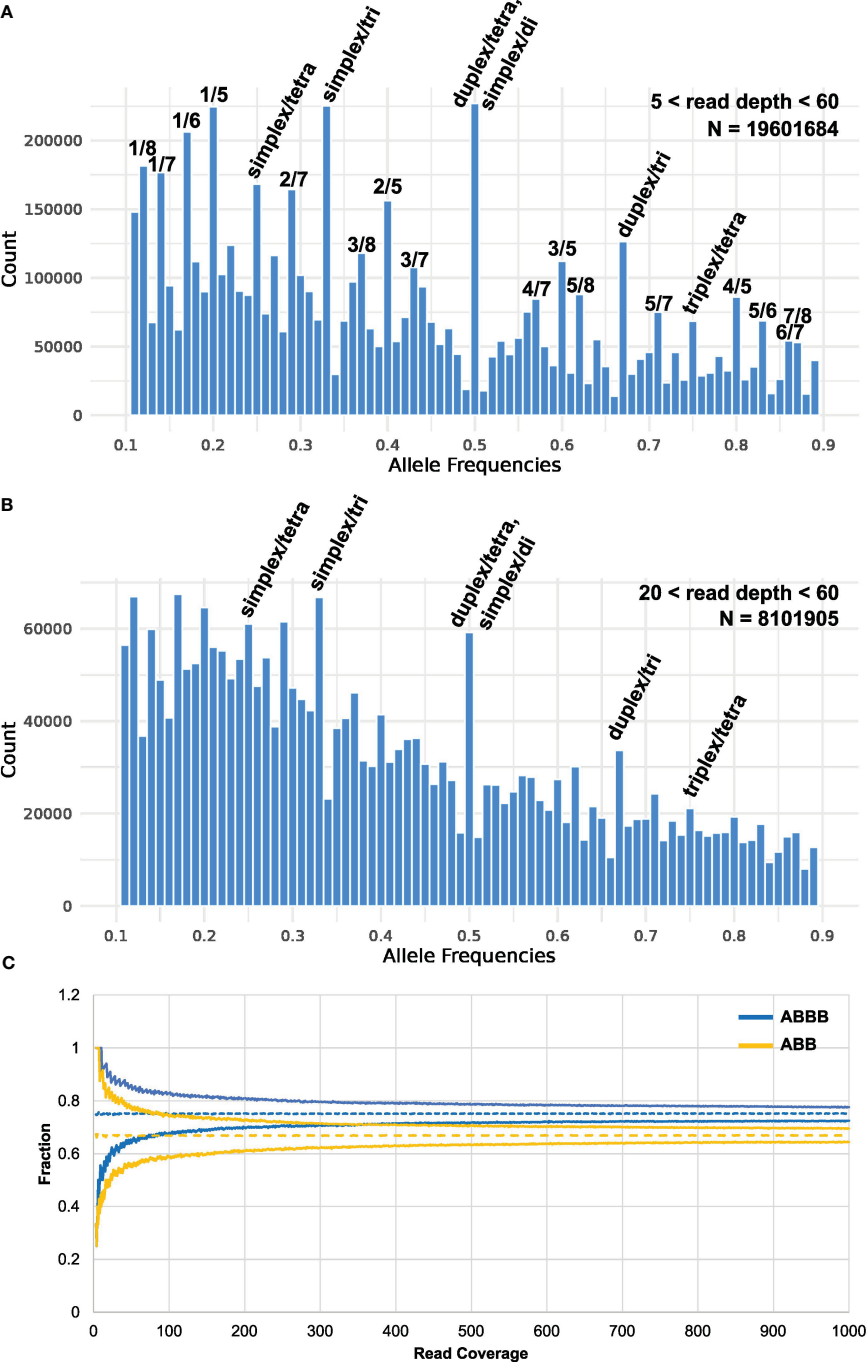
Histogram of the observed allele frequency ratio across all heterozygous genotypes (i.e., 0.1< ratio< 0.9, bin width = 0.01) of the MASPOT genotypes for read depth of **(A)** 5–60 (*N* = 19601684 post-filtering: 755 samples, 31,007 SNPs) and **(B)** 20–60 (*N* = 8101905: 755 samples, 10,731 SNPs). Peaks corresponding to discrete heterozygous states are marked. **(C)** Confidence intervals (95%) of simulated allele frequency ratio distributions of heterozygous tetraploid (ABBB, 0.25 A/0.75 B) and triploid (ABB, 0.33 A/0.67 B) states as a function of sequencing depth.

It has previously been found that calling tetraploid allele dosages from sequence-based genotyping data is challenging from low-sequence coverage studies (Li et al., 2014), and it has been suggested that a coverage of 60–80× is appropriate to secure concordance between sequence-based genotyping calls and KASP analysis (Uitdewilligen et al., 2013). However, the recent finding that elite autotetraploid potato has an observed average ploidy of 3.2 across all loci (Sun et al., 2022) complicates this problem even further. An even higher resolution is demanded for distinguishing between all the possible heterozygous genotypes of both tetraploid and triploid loci, which can be expected to occur at high frequencies: AAAB, AABB, ABBB, AAB, and ABB. When plotting the observed frequency ratio of heterozygous SNPs across all MASPOT genotypes, peaks corresponding to 0.25, 0.33, 0.5, 0.67, and 0.75 are apparent (**Figure 1A**), supporting the finding that many loci deviate from a tetraploid state. Furthermore, peaks are observed at 0.125, 0.375, 0.625, and 0.875, which seem to suggest octoploid heterozygous loci, as well as peaks at 0.167 and 0.833 would suggest hexaploid heterozygous loci. We speculate that these may stem from the mapping of duplicated gene loci in the genotyped cultivars to a single locus in the reference. In such a case, the observed octoploid distribution is expected for the mapping of two tetraploid loci, and the observed hexaploid distribution for the mapping of two triploid loci. Following this notion, another two loci scenario (tetraploid at one locus and triploid at another) would give rise to peaks in the histogram at 0.143, 0.286, 0.428, 0.571, 0.714, and 0.857 and, indeed, peaks can be observed at these positions. A diploid locus and a triploid locus mapping to one locus would give rise to peaks at 0.2, 0.4, 0.6, and 0.8, which is also observed. Overall, this is in agreement with the finding by Sun et al. (2022) that allele copy numbers of 1–4 were observed across loci in the Otava genome. On the other hand, the read coverage of SNPs included in the analysis was in the range of 5–60, with a mean of 19.2 and a median of 14.6 (**Supplementary Figure S1**). Therefore, we cannot exclude that the observed structure in the data stems from binomial sampling at low sampling depth which is also expected to give rise to structure in the data. However, the frequency ratios of the subset of markers with the highest coverage (between 20 and 60) also display the distinct 0.25, 0.33, 0.5, 0.67, and 0.75 peaks representing the cooccurrence of loci of varying ploidy in the potato genome (**Figure 1B**), supporting that the observed non-tetraploid ratios are not caused by sampling errors. Regardless, it is apparent from **Figures 1A, B** that, with the read coverage in our study, the main distributions, representing triploid and tetraploid loci, are overlapping and hence, there is insufficient resolution to reliably determine the per-loci ploidy state using the observed data directly for most loci. Therefore, we sought to simulate how the ability to distinguish between the “worst case scenario”—ABB and ABBB is developing with sequence coverage by simulating a random sampling of N depth from a distribution of 0.33 A/0.67 B and 0.25 A/0.75 B, respectively. In **Figure 1C**, the 95th and 5th percentiles of the simulated distribution of SNP frequency ratios are shown for tetraploid and triploid loci, respectively. It is obvious, that even in this ideal case in the absence of sequencing errors, extreme sequence depth in excess of ~320X is needed to distinguish between a heterozygous triploid and a heterozygous tetraploid state with 95% confidence. This underscores that it is not practically feasible to distinguish between tetraploid and triploid loci with sequence-based genotyping methods, at least with the current cost of sequencing technologies. Similarly, Grandke et al. (2016) found that heterozygous states could not successfully be called in hexaploid chrysanthemum, because allele signals did not segregate into distinguishable clusters—an assumption of the applied genotype caller SuperMASSA. The poor resolution of heterozygous signals in polyploids, an issue of escalating complexity with increased ploidy (de Bem Oliveira et al., 2019), indicates that the continuous genotypes are currently the best option for genotyping polyploids. This adds to the relevance of investigating whether calling discrete genotypes affects GP and GWAS performance in potatoes compared to continuous genotype values.

### 2.2 Genomic prediction of five single traits yields similar results irrespective of calling allele dosage

**Figure 2:**
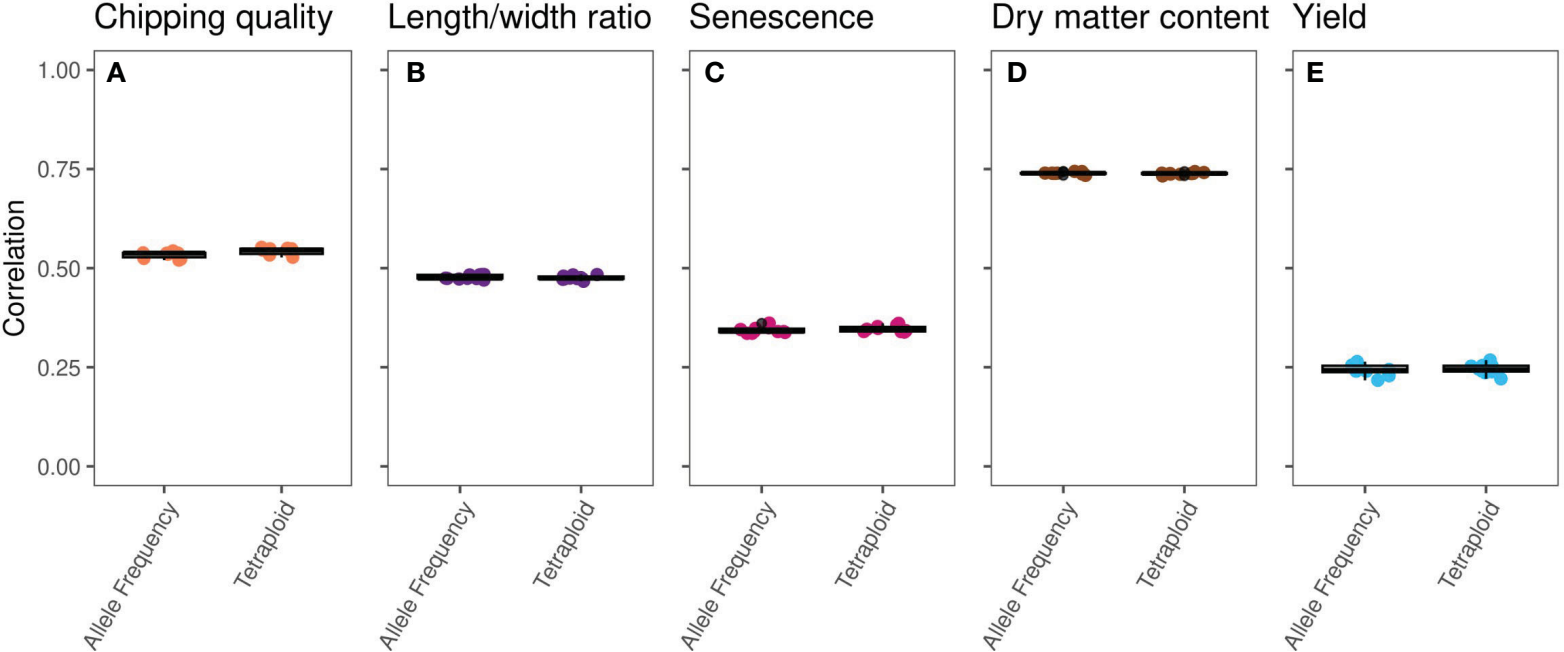
Boxplots of single-trait GBLUP prediction correlation coefficients, calculated for 10 repeats of eightfold random cross validation, between observed phenotype and genomic estimated breeding values (GEBVs) of the MASPOT panel on an identical set of SNPs called as either observed allele frequencies (left) or tetraploid genotypes (right) for the five traits: **(A)** chipping quality, **(B)** length/width ratio, **(C)** senescence, **(D)** dry matter content, and **(E)** yield.

Single-trait GP of the five traits using the true marker dosage (continuous allele frequency SNPs) and forced tetraploid markers performed almost identically (**Figure 2**, **Supplementary Figure S2**, **Table 1**), only chipping quality (with the highest amount of missing phenotype data [30%]) (*p* = 1.3*10^−8^) and senescence (*p* = 0.018) being modeled to improved mean correlation coefficients using the tetraploid compared to continuous SNPs. However, the numerical increase of the mean correlation is only a diminutive rise from 0.534 to 0.543 for chipping quality and from 0.343 to 0.347 for senescence. This could be a result of a very small trait-dependent response to the genotyping method, similar to previous reports on diploidization in potato (Gemenet et al., 2020). However, the difference in mean correlation for chipping quality could also be attributable to the effectively smaller training population for this trait, due to the large amounts of missing phenotypic data. Overall, based on these results genomic prediction is largely insensitive to calling genotypes as continuous or using a tetraploid model.

**Table 1:** Mean prediction Pearson correlation coefficients and prediction biases between GEBVs and observed phenotypic values for 10 repeats of random eightfold cross-validated GBLUP genomic predictions of allele frequency (AF) and tetraploid (T) markers on the MASPOT panel.

	Chipping quality		Length/width ratio		Senescence		Dry matter content		Yield	
	AF	T	AF	T	AF	T	AF	T	AF	T
**Correlation**	0.534	0.543	0.477	0.476	0.343	0.347	0.740	0.739	0.243	0.245
**Prediction bias**	1.040	1.066	1.011	1.014	0.997	1.001	1.024	1.031	0.906	0.924

It has already been shown for loci with relatively low read coverage that it is not possible to distinguish between all three heterozygous states of an autotetraploid (Uitdewilligen et al., 2013; Li et al., 2014; Byrne et al., 2020), and our simulation shows that it is practically unattainable to confidently discriminate between heterozygous triploid and tetraploid loci with coverage below <320×. Irrespective of this, forcing tetraploid SNP calls in our case with loci of read depth greater than 5× and smaller than 60× did not affect model performance compared to using the observed allele frequencies—effectively validating previous genomic prediction model results based on tetraploid SNP calls in potato. Notably, while high coverage is needed to confidently call heterozygous tetraploid genotypes and discriminate ploidy, using read depths >60× to determine allele frequencies has been reported to result in changes in allele frequency variances (Ashraf et al., 2016), possibly influenced by signal from abundant repetitive elements, why using loci with extremely high read coverage is generally not recommended or employed. Results from autotetraploid blueberry further indicates that for accurate predictions using continuous genotypes, it is sufficient to use low-mid read depths of 6–12× compared to 60× (de Bem Oliveira et al., 2020).

We previously established that prioritizing continuous SNPs according to type (i.e., nsSNP, sSNP, ncSNP, or all combined) also did not affect GP model performance for most of the traits analyzed here (except yield) (Aalborg et al., 2024). Likely, the reasons for the invariability of the prediction performances observed in these two cases are similar and both related to the LD decay of the potato genome and the distance between the anonymous marker and the causal variant. With an estimated LD block size of 0.6–1.5 Mb and 2–5 Mb in introgressed genome regions (Vos et al., 2017), it can be extrapolated that, for an LD block size of 1 Mb, all markers in a 500 kb window on either side of a causal variant can be used as a reasonably reliable marker, that is, with a redundant SNP set of ~31k genome-wide markers there is likely to be found correctly called variants in LD with causal trait variants when using a tetraploid model. Hence, the linkage effect is likely sufficiently large to predominate over any erroneous signals of miscalled allele dosages. While we did not examine the effect of read depth on prediction accuracy in potato, it is plausible that a similar effect could support the use of low read depth data. As genotype quality is proportional to read depth, the effective pool of accurately sampled SNPs is lowered with decreased sequencing depth but is likely still sufficiently large to include reliable LD block markers. Previous results have shown that GP models are resilient to marker reductions in a trait-dependent manner in potato, but that across several traits only 1–10k markers of read depth between 5–60x were required to reach optimal model performance (Aalborg et al., 2024).

### 2.3 Genome-wide association studies yield similar results irrespective of calling allele dosage

**Figure 3:**
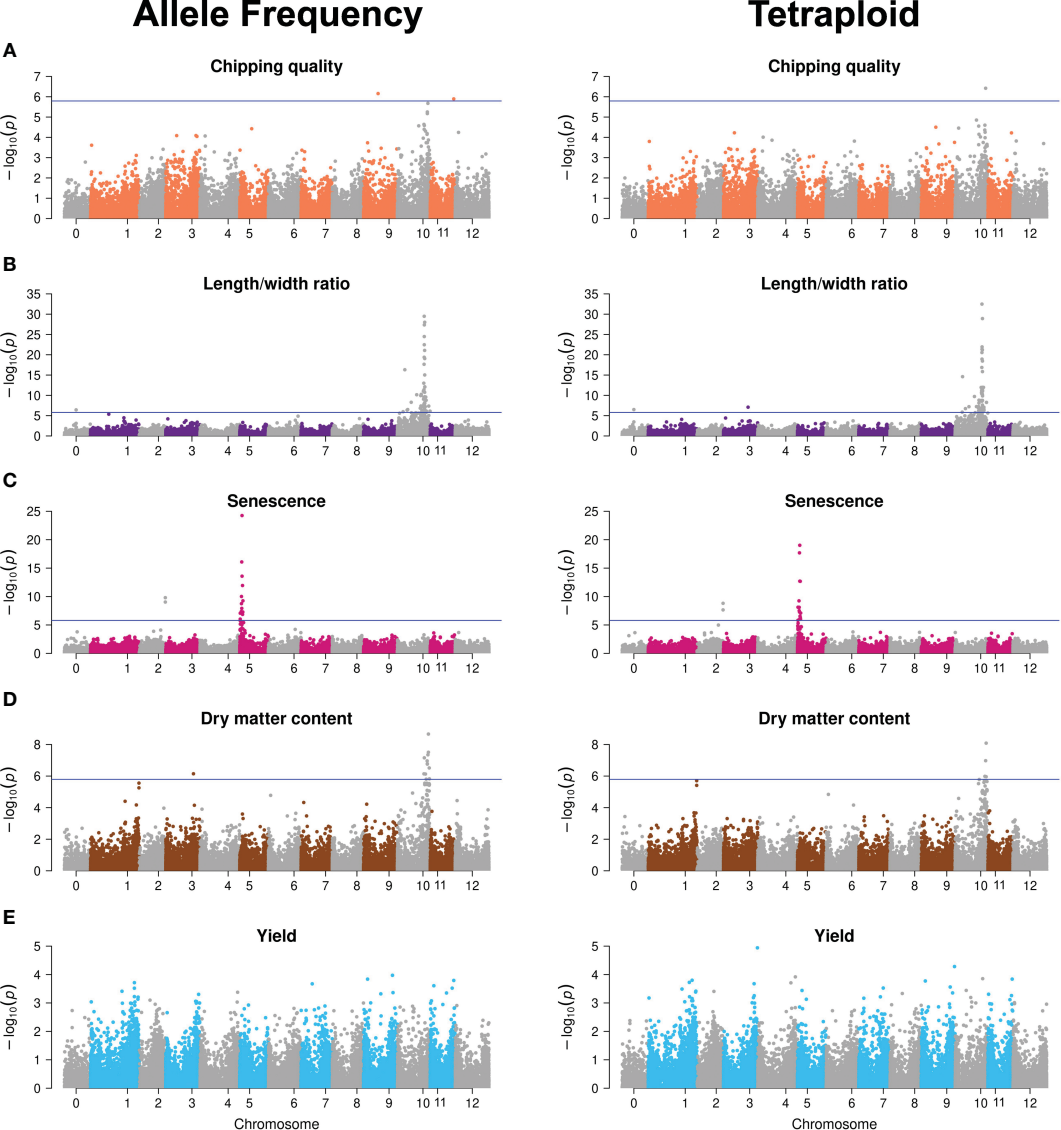
Manhattan plots of −log_10_ (*p*-value) GWAS results of the allele frequency markers (left) and tetraploid markers (right) for the five traits: **(A)** Chipping quality, **(B)** length/width ratio, **(C)** senescence, **(D)** dry matter content, and **(E)** yield. Chromosome 0 is a bin of unanchored contigs and scaffolds in the reference genome. The horizontal line indicates the genome-wide Bonferroni-corrected significance threshold.

An elaborate description of the major QTLs identified is presented in (Aalborg et al., 2024). The GWAS analyses with tetraploid SNPs reproduced the major QTLs found with continuous genotypes (**Figure 3**; **Supplementary Files S1, S2**). Differences constituted possible minor QTLs, major QTL significance, and therefore also the number of significant SNPs constituting the major QTL signals. The latter is observed for dry matter content, where continuous SNPs generate a major QTL signal on chromosome 10 from 48 to 58 Mb constituted of 13 significant markers, whereas the chromosome 10 signal is reduced to four SNPs in the 53–56 Mb range when using tetraploid SNPs. For major trait QTLs, the tetraploid SNPs hence hold sufficient true information about the trait genetic variation to accurately map those QTLs, albeit with slightly reduced significance, observed as variation in signal intensity in terms of both range and amplitude. However, for the identification of candidate minor QTL effects, a differential outcome is observed, underscoring the need for validation of such QTLs. The higher sensitivity of GWAS performance compared to GP is in agreement with what we previously found when analyzing the effect of marker type (Aalborg et al., 2024).

This indicates that, while the overall amount of additive genetic variance explained is similar across the two genotyping methods, as it is captured to the same level by the genomic prediction model, continuous genotypes contribute alternative information when resolved to chromosomal signal location. We speculate that this may vary across traits for two reasons. First, this could reflect uneven dispersion of the gene copy number variations, a likely result of progressive phase deletions during successive meiotic recombination events, effectively generating a locus-dependent response to the genotyping method. In regions where the local LD-decay is low due to hot spots for recombination events, the local concentration of true markers becomes site-dependent using the tetraploid model. Secondly, our inability to reliably call the missing phase for any locus and, hence, the potential haploinsufficiency effects of a triploid versus tetraploid site becomes cryptic. If there indeed is an effect, it is not specially specified as a potential feature in the statistical models and, hence, we have no marker for the specific difference of triploid/tetraploid and therefore it is not fully included in the analysis with either genotyping method.

### 2.4 Consequences for breeding

The resilience of population/family structure-corrected GP models to marker reduction (Sverrisdóttir et al., 2018; Selga et al., 2021; Aalborg et al., 2024), their indifference to marker type filtration (Aalborg et al., 2024), and, as seen here, their general tolerance to allele dosage parametrization (Endelman et al., 2018; de Bem Oliveira et al., 2019; Matias et al., 2019; Gemenet et al., 2020; Yadav et al., 2024) all suggest that efforts such as SNP pruning, high-depth sequencing (de Bem Oliveira et al., 2020), and so forth can neither notably improve model performance in potato for complex, quantitative traits such as yield nor detection of single major QTL traits such as senescence and length/width ratio beyond a plateau level, which is reached with relatively shallow analyses. Rather, future efforts in increasing model performance should be directed toward increasing the size of phenotype/genotype datasets and capturing the non-additive genetic variation of traits, for example, dominance, epistasis [though this has generated mixed results in previous studies (Su et al., 2012; Endelman et al., 2018; Wilson et al., 2021)], pleiotropy, as well as gene–environment interactions. Such studies are further complicated by the fact that a full tetraploid model is not appropriate for modeling these effects across all loci. To capture the effects of haploinsufficiency of phase deletions these should also be specified in models for continuous markers capable of capturing ploidy variations.

In practice, as documented in this study, using a tetraploid model for calling SNPs in potato had largely no effect on genomic predictions compared to using observed allele frequencies and only resulted in very minor variations in GWAS results. This constitutes a relief for existing genomics-assisted breeding programs since existing GP models that are used need not be remade. Particularly, because determining gene allele dosage from sequence data directly is practically infeasible. Nonetheless, a general conservative statistical modeling approach is generally advocated where models are kept as simple as possible unless an explicit argument for more complicated models is presented and the differential effects documented. This dictates that tetraploid SNP calls should not be used, but rather the observed allele frequencies computed directly from sequencing data as continuous genotypes. As results from (de Bem Oliveira et al., 2020) indicates that low-mid sequencing depth is sufficient for accurate prediction with continuous genotypes, this does not necessarily entail inflated genotyping costs (Ferrão et al., 2021). In extension, pseudo-diploidization is also discouraged based on results from (Endelman et al., 2018), showing deflated prediction accuracies with this parametrization. Using continuous genotypes also entails a reduction in computational time, as the genotype calling step is circumvented by calculating allele frequencies directly from sequencing data (de Bem Oliveira et al., 2019).

## 3 Conclusions

In conclusion, the impact of using continuous SNP markers based on allele frequency from read coverage compared to enforcing a tetraploid dosage model on potato was not found to impact the performance of genomic prediction models but did result in minor variations of signal amplitude in GWAS, hence effecting minor QTL detection. Inspection of the distribution of allele frequency ratios of the continuous biallelic markers revealed peaks consistent with the ploidy variation previously reported for the “Otava” cultivar. Simulation data also revealed that extreme read depths of >320× are required to accurately distinguish between heterozygous triploid and tetraploid states, in addition to known high read depth requirements to separate the three heterozygous tetraploid states. While results did not indicate that genomic prediction performance is affected by using a tetraploid model for genotype calling, we encourage using continuous markers for statistical modeling of potato to represent most accurately the true genomic architecture of the plant crop.

## 4 Methods

Statistical analyses were processed using R Statistical Software (v4.3.2) (R Core Team, 2023) in RStudio (v2023.9.1.494) (Posit team, 2023) and graphics were prepared using the ggplot2 package (v3.4.4) in R (Wickham, 2016) unless otherwise stated.

See Aalborg et al. (2024) for detailed description and analysis of plant material, phenotype distributions, and population structure in the MASPOT panel.

### 4.1 Genotyping

Genotyping of the MASPOT panel was performed by genotyping-by-sequencing (GBS) as described in (Sverrisdóttir et al., 2017). GBS libraries were prepared following a protocol adapted from (Elshire et al., 2011). Illumina 5′ and 3′ adaptors for sequencing were designed for a 96-multiplexing system. Leaf tissue DNA was extracted and digested with *ApeKI*, the fragments were ligated to adaptors and pooled in 96-plex libraries, purified, and amplified by PCR. The libraries were then sequenced on a HiSeq 2000 (Illumina, San Diego, USA) with single-read sequencing (100 bp). Each 96-plex library was sequenced on three channels of a flow cell.

### 4.2 Simulation of SNP ratio data

Custom bash scripts (**Supplementary File S3**) were used to simulate a random sampling of N depth at 2,500 loci from parental distributions of ABB or ABBB, respectively. N was varied from 3 to1,000. The SNP ratio average, the 95th and 5th percentiles was calculated from either case and plotted using Microsoft Excel. The crossing of the 5th percentile of the ABBB case with the 95th percentile of the ABB case was taken as 95% confidence threshold.

### 4.3 Filtering and mapping sequence data and SNP calling

The sequenced reads were processed as described in (Sverrisdóttir et al., 2017). The reads were demultiplexed, trimmed, and mapped onto the reference genome of double monoploid *S. tuberosum* Group Phureja DMv4.03 (Sharma et al., 2013). Tetraploid SNPs were called using the Genome Analysis Toolkits (McKenna et al., 2010) UnifiedGenotyper tool with ploidy set to 4, a minimum phred-scaled confidence threshold of 50 for variant calling and of 20 for variant omission (and filtered with LowQual for below the calling threshold), cf (Sverrisdóttir et al., 2017). SNPs were filtered to a minimum root mean squared quality of 30 and reduced to only biallelic variants. In addition to the tetraploid variant call, observed allele frequency estimated directly from sequencing data was also used as genotyping method, cf. (Ashraf et al., 2016) to accommodate the gene copy number variation across loci in the potato genome (Sun et al., 2022). The allele frequencies were determined as the ratio between allele counts of the alternative allele and the total allele count, giving a continuous allele ratio between 0 and 1, but not constricted to tetraploid dosages (**Equation 1**).


(1) $$AF=\frac{AC_{alt}}{AC_{ref}+AC_{alt}}$$

This provided two sets of genotypes for the same SNPs, that is, continuous genotypes and tetraploid genotypes. The five possible tetraploid genotypes [AAAA, AAAB, AABB, ABBB, or BBBB] were recoded to [0, 1, 2, 3, 4] allele dosages. Minor alleles frequency (MAF) was calculated from read coverage, and SNPs were filtered to an MAF of 1% (i.e., mean allele frequency<0.99 and >0.01), missing rate of maximum 50%, and finally filtered to read coverage >5 at all positions, introducing a missing genotype for low-quality positions in individual samples. Based on the continuous genotypes, the SNPs were further filtered to SNPs with read coverage between 5 and 60 and samples were filtered to clones with <70% missing data. A final SNP set of ~31k positions of the filtered SNPs spanning the entire genome was used for further analysis. The SNP set originates from (Aalborg et al., 2024) and consists of 31,032 SNPs that are approximately evenly composed of non-synonymous SNPs, synonymous SNPs, and non-coding SNPs (**Supplementary Figure S3**, **Supplementary File S4**). Both the continuous and tetraploid genotypes were reduced to that SNP set. SNPs that were monomorphic in either genotype matrix were filtered from both sets, yielding two final genotype matrices of 31,007 markers. Finally, the tetraploid genotypes were recoded from [0, 1, 2, 3, 4] to [0, 0.25, 0.50, 0.75, 1] to match the continuous genotype matrix format.

### 4.4 Statistical analyses

#### 4.4.1 Genomic prediction models

Single-trait standard additive GBLUP was used to directly estimate GEBVs using the genomic relationship (G) matrix (Meuwissen et al., 2001) (**Equation 2**):


(2) y=1μ+g+e

, where y is a vector of observed phenotypes, *µ* is the mean, g is a vector of random genomic breeding values following distribution $g\;∼\left(N,\;G{\text{σ}}_{g}^{2}\right)$ , where G is the genomic relationship matrix and ${\text{σ}}_{g}^{2}$ is the genetic variance of the model, and e is a vector of residuals with distribution $e\;∼\left(N,\;I{\text{σ}}_{e}^{2}\right)$ , where I is an identity matrix and ${\text{σ}}_{e}^{2}$ is the residual variance.

Either marker set was corrected for missing data following the correction, $w_{i}$ , described by VanRaden (2008) (**Equation 3**).


(3) $$w_{i}=\sqrt{\frac{\sum p_{k}\left(1-p_{k}\right)\;over\;all\;loci}{\sum p_{k}\left(1-p_{k}\right)\;over\;only\;non-missing\;loci}}$$

, where $p_{k}$ is the mean genotype at locus *k*. The genotype matrices were centered and adjusted for missing values according to (Ashraf et al., 2016), and then missing genotypes were imputed by mean imputation (means set to zero) (**Equation 4**). A total of 16.3% of markers were imputed.


(4) $${\text{Z}}_{ik}=\left({\text{X}}_{ik}-p_{k}\right)\cdot w_{i}$$

, where Z is the genotype matrix, $X_{ik}$ is the genotype in family *i* at locus *k*. From Z , the G -matrices were computed using global scaling cf. the VanRaden (2008) method 1 with an adjustment for tetraploidy (**Equation 5**).


(5) $$\text{G}=\frac{\text{Z}{\text{Z}}^{\prime }}{0.25\sum p_{k}\left(1-p_{k}\right)}$$

, where $0.25\sum p_{k}\left(1-p_{k}\right)$ is the cumulative genotypic variance as well as the average $ZZ^{\prime }$ diagonal.

All GBLUP models were computed using the BGLR package (v1.1.0) in R (Pérez and de los Campos, 2014) with 12,000 iterations, 2,000 burn-in, and default priors. Each analysis was performed with eightfold random cross-validation in 10 repeats of different cross-validation groupings. The accuracy of the GEBVs was determined as the Pearson correlation coefficient between the GEBVs and the observed phenotypes (**Equation 6**):


(6) r(GEBV:y)

The samples of 10 correlation coefficients obtained were compared pairwise for allele marker frequency and tetraploid genotypes for each of the five traits by paired sample *t*-tests of the Fisher *r*-to-*z*-transformed correlation coefficients with a 0.05 significance threshold.

Prediction bias was calculated as the slope (β) of the linear regression between the observed phenotypes and GEBVs, where β = 1 indicates no bias, β< 1 indicates that extremely high (low) GEBVs are over-(under-)estimated compared to the realized phenotypes, and opposite for β > 1 (Luan et al., 2009).

#### 4.4.2 Genome-wide association studies

Single-trait GWAS was performed using the tetraploid and the allele frequency genotypes for each of the five traits by single-marker regression in R using the regress package (v1.3.21) (Clifford and McCullagh, 2006, 2020) (**Equation 7**):


(7) $$\text{y}=1\text{μ}+{\text{X}}_{i}{\text{β}}_{i}+\text{g}+\text{e}$$

where y is a vector of observed phenotypes, $X_{i}$ is a vector of SNP genotypes at the *ith* position and ${\text{β}}_{i}$ is the corresponding additive effect, g is a vector of random genomic breeding values following distribution $g\;∼\left(N,\;G{\text{σ}}_{g}^{2}\right)$ , where G is the genomic relationship matrix, and e is a vector of residual effects with distribution $e\;∼\left(N,\;I{\text{σ}}_{e}^{2}\right)$ . For each chromosome, a G -matrix was calculated based on the variants on 11 of the 12 chromosomes, excluding the chromosome encoding the *ith* position SNP to avoid including the SNP in the model twice (Kristensen et al., 2018). The genomic relationship matrix was then used to correct for population structure in the diallel cross panel. Additional population structure can arise from recurrent genetic variance in the pedigrees of elite cultivars as a result of clonal propagation through seed potatoes. This can result in overdispersion of the test statistics in association analyses (Devlin et al., 2001), inflating the associations and generating false positive associations. The *p*-values were corrected for this using the genomic inflation factor, ${\text{λ}}_{gc}$ (Hinrichs et al., 2009; Kristensen et al., 2018). Inflation factors were calculated for each trait and both tetraploid and allele frequency genotypes.


(8) $$\begin{array}{l} {\text{λ}}_{gc}=\frac{median\left({\text{χ}}^{2}\right)}{Q_{{\text{χ}}^{2}}^{-1}\left(0.5,1\right)},\\ {\text{χ}}^{2}=Q_{{\text{χ}}^{2}}^{-1}\left(P,1\right) \end{array}$$

The inflation factor was computed as the median value of the ${\text{χ}}^{2}$ -statistics of the SNPs, converted from each *p*-value (*P*) using the inverse cumulative distribution function (CDF) of the ${\text{χ}}^{2}$ -distribution, $Q_{{\text{χ}}^{2}}^{-1}$ , with 1 degree of freedom, divided by the expected median, assuming no association between the SNP and trait phenotype, that is, the ${\text{χ}}^{2}$ -statistic of the 50th percentile (**Equation 8**). For ${\text{χ}}^{2}>1$ , indicating systematic effects not captured by the model, the *p*-values were corrected by dividing the SNP ${\text{χ}}^{2}$ -statistic by ${\text{λ}}_{gc}$ and the result converted to corrected *p*-values using the CDF of the ${\text{χ}}^{2}$ -distribution with 1 degree of freedom (**Equation 9**).


(9) $$P_{corrected}=1-Q_{{\text{χ}}^{2}}\left(\left(\frac{{\text{χ}}^{2}}{{\text{λ}}_{gc}}\right),1\right)$$

Bonferroni correction was used to control for false positive associations with a false discovery rate of p<0.05/N , where 0.05 is the significance threshold and N is the number of markers analyzed. QQ and Manhattan plots were generated using the qqman package (v0.1.9) in R (Turner, 2018).

## Data availability statement

The datasets presented in this study can be found in online repositories. The name of the repository and accession number can be found below: 10.5281/zenodo.10664896.

## Author contributions

TA: Conceptualization, Formal analysis, Investigation, Methodology, Project administration, Visualization, Writing – original draft, Writing – review & editing. KN: Conceptualization, Formal analysis, Funding acquisition, Investigation, Methodology, Project administration, Supervision, Visualization, Writing – original draft, Writing – review & editing.
