# Supplementary material for: To be or not to be tetraploid—the impact of marker ploidy on genomic prediction and GWAS of potato
Source: Front Plant Sci. 2024 Jul 30;15:1386837. doi: 10.3389/fpls.2024.1386837 (PMC11319270; doi:10.3389/fpls.2024.1386837)
Supplement: Supplementary file 2 [file DataSheet_2.docx]

Supplementary Materials

To Be or Not to Be Tetraploid – The Impact of Marker Ploidy on Genomic Prediction and GWAS of Potato

Trine Aalborg*, Kåre Lehmann Nielsen

*** Correspondence:** Trine Aalborg: traa@bio.aau.dk

# Supplementary Figures


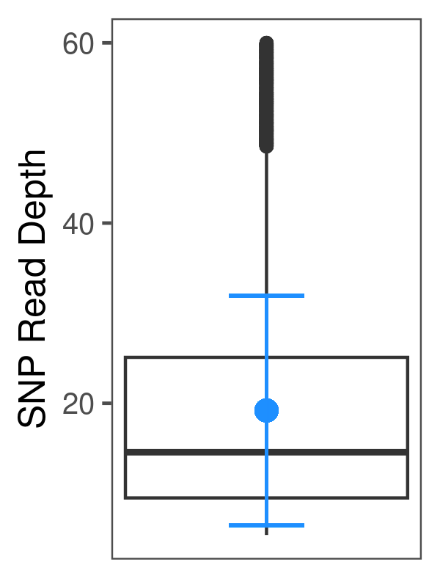


**Supplementary Figure S1.** Boxplot of SNP read coverage for the 31,007 SNPs called as either tetraploid or observed allele frequency. The mean read coverage and is plotted as a blue dot with whiskers of one standard deviation in blue.


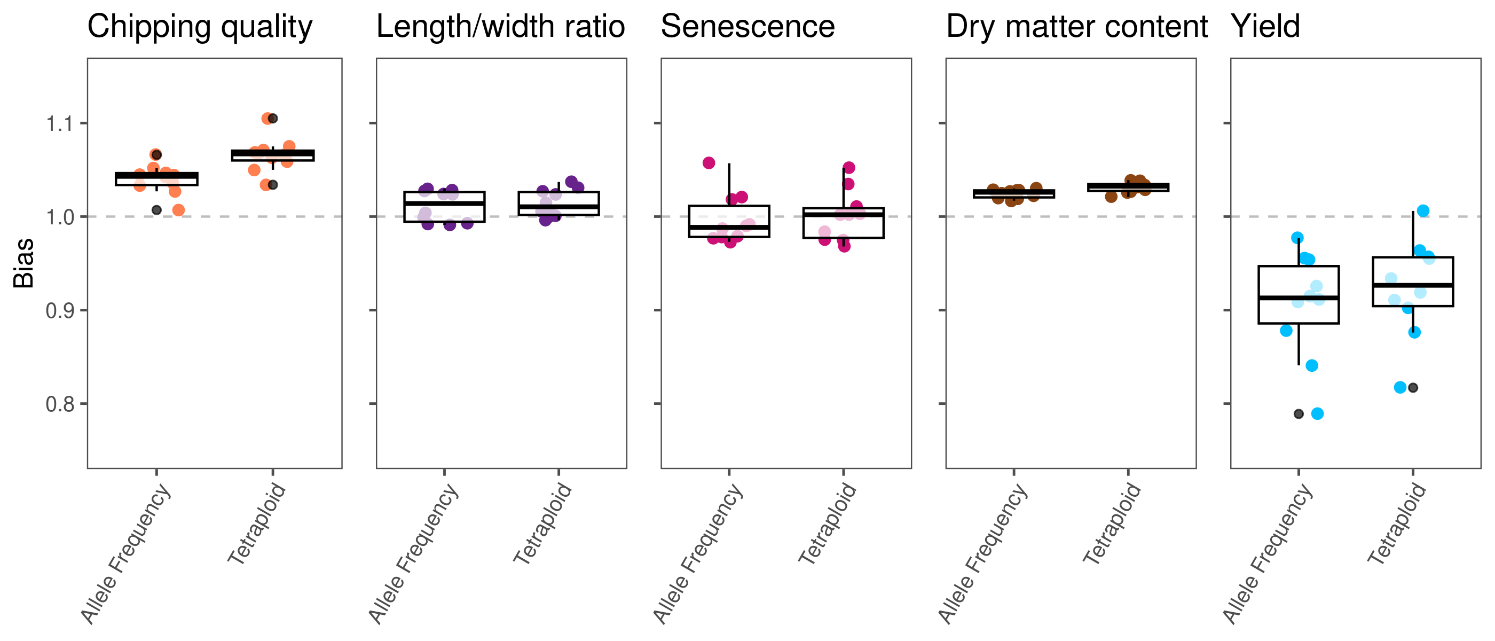


**Supplementary Figure S2.** Boxplots of prediction bias of single-trait GBLUP predictions of the MASPOT panel for the five traits chipping quality, length/width ratio, senescence, dry matter content, and yield, calculated for 10 repeats of random 8-fold cross-validation, on an identical set of SNPs called as either allele frequency (left) or tetraploid genotypes (right). The dashed gray line of bias = 1 indicates no prediction bias.


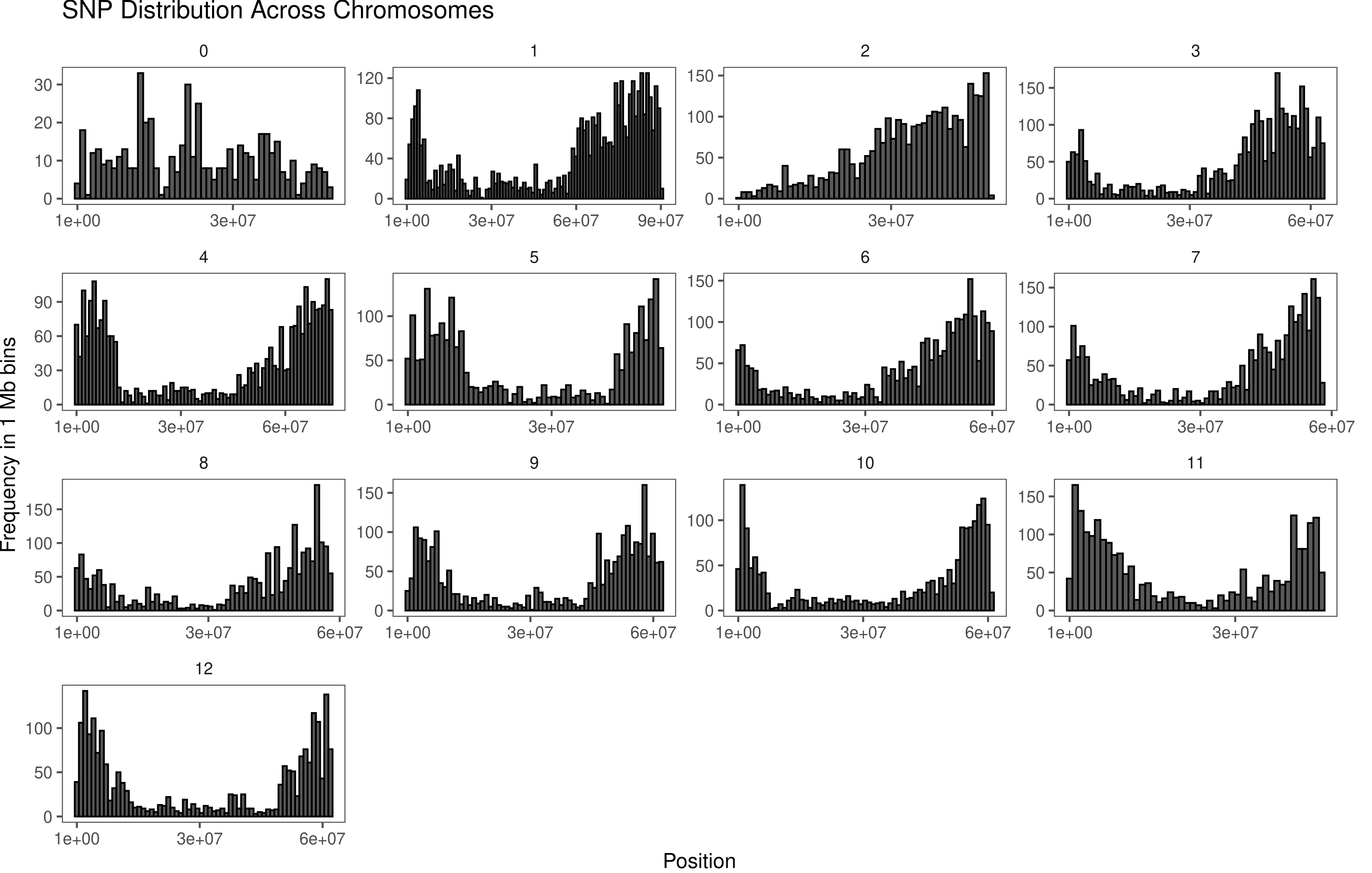


**Supplementary Figure S3.** Histograms of marker density in 1 Mb windows along the 12 potato chromosomes for the 31,007 SNPs called as either tetraploid or observed allele frequency. Chromosome 0 is pseudomolecules.
