# Supplementary material for: To be or not to be tetraploid—the impact of marker ploidy on genomic prediction and GWAS of potato
Source: Front Plant Sci. 2024 Jul 30;15:1386837. doi: 10.3389/fpls.2024.1386837 (PMC11319270; doi:10.3389/fpls.2024.1386837)
Supplement: Supplementary file 5 [file Table_3.docx]

#tetratriplex_coverage_generator.bash

#generate snp observations based on triduplex and tetraduplex distributions.

mkdir temp

#Tetraploid - generate random integer betwen 0 and 3

awk 'BEGIN {for(i=1;i<=40000;i++) {print int(rand()*4)}}' > tetra_random_numbers.temp

#covert to bases

awk '{if($1==0) {print "A"} else {print "B"}}' < tetra_random_numbers.temp | tr -d '\n' > random_string.temp

#cat random_string.temp

#sample strech based on read coverage

string=$(cat random_string.temp)

i=3

> temp/SNP_strings_cov$i

while [ $i -le 250 ]

do

echo "generating strings with SNP read coverage; "$i

j=1

while [ $j -le 2500 ]

do

start=$RANDOM

# end=$(($start+$i))

# echo "random variable: "$start

# echo "read coverage: "$i

# echo "random base: ${string:$start:$i}"

echo "${string:$start:$i}" >> temp/SNP_strings_cov$i.temp

((j++))

done

#calculating SNP ratio

# echo $i" pass"

echo $i > temp/SNP_ratio_simulation_$i

awk -F '' '{print NF}' temp/SNP_strings_cov$i.temp > temp/NF_$i

# echo "test1"

awk -F 'B' '{print NF-1}' temp/SNP_strings_cov$i.temp > temp/B_$i

# echo "test2"

paste temp/NF_$i temp/B_$i | awk '{print $2/$1}' >> temp/SNP_ratio_simulation_$i

((i++))

done

#parsing to single formatted files

paste temp/SNP_ratio_simulation_* > tetra_SNP_ratio_simulation_final.txt

#

#

#triduplex_coverage_generator.bash

#generate snp observations based on triduplex and tetraduplex distributions.

mkdir temp

#Triploid - generate random integer betwen 0 and 2

awk 'BEGIN {for(i=1;i<=40000;i++) {print int(rand()*3)}}' > Tri_random_numbers.temp

#covert to bases

awk '{if($1==0) {print "A"} else {print "B"}}' < Tri_random_numbers.temp | tr -d '\n' > random_string.temp

#cat random_string.temp

#sample strech based on read coverage

string=$(cat random_string.temp)

i=3

> temp/SNP_strings_cov$i

while [ $i -le 250 ]

do

echo "generating strings with SNP read coverage; "$i

j=1

while [ $j -le 2500 ]

do

start=$RANDOM

# end=$(($start+$i))

# echo "random variable: "$start

# echo "read coverage: "$i

# echo "random base: ${string:$start:$i}"

echo "${string:$start:$i}" >> temp/SNP_strings_cov$i.temp

((j++))

done

#calculating SNP ratio

# echo $i" pass"

echo $i > temp/SNP_ratio_simulation_$i

awk -F '' '{print NF}' temp/SNP_strings_cov$i.temp > temp/NF_$i

# echo "test1"

awk -F 'B' '{print NF-1}' temp/SNP_strings_cov$i.temp > temp/B_$i

# echo "test2"

paste temp/NF_$i temp/B_$i | awk '{print $2/$1}' >> temp/SNP_ratio_simulation_$i

((i++))

done

#parsing to single formatted files

paste temp/SNP_ratio_simulation_* > tri_SNP_ratio_simulation_final.txt
